# Air pollution relevance analysis in the bay of Algeciras (Spain)

**DOI:** 10.1007/s13762-022-04466-4

**Published:** 2022-09-12

**Authors:** M. I. Rodríguez-García, J. González-Enrique, J. A. Moscoso-López, J. J. Ruiz-Aguilar, I. J. Turias

**Affiliations:** 1 Department of Computer Science Engineering, PolytechnicSchoolofEngineering, University of Cádiz, Algeciras, Spain; 2 Department of Industrial and Civil Engineering, PolytechnicSchoolofEngineering, University of Cádiz, Algeciras, Spain

**Keywords:** Air pollution, Regression analysis, Relevant variables, Relative risk, Spatial behavior

## Abstract

The aim of this work is to accomplish an in-depth analysis of the air pollution in the two main cities of the Bay of Algeciras (Spain). A large database of air pollutant concentrations and weather measurements were collected using a monitoring network installed throughout the region from the period of 2010–2015. The concentration parameters contain nitrogen dioxide (NO_2_), sulphur dioxide (SO_2_) and particulate matter (PM_10_). The analysis was developed in two monitoring stations (Algeciras and La Línea). The higher average concentration values were obtained in Algeciras for NO_2_ (28.850 µg/m^3^) and SO_2_ (11.966 µg/m^3^), and in La Línea for PM_10_ (30.745 µg/m^3^). The analysis shows patterns that coincide with human activity. One of the goals of this work is to develop a useful virtual sensor capable of achieving a more robust monitoring network, which can be used, for instance, in the case of missing data. By means of trends analysis, groups of equivalent stations were determined, implying that the values of one station could be substituted for those in the equivalent station in case of failure (e.g., SO_2_ weekly trends in Algeciras and Los Barrios show equivalence). On the other hand, a calculation of relative risks was developed showing that relative humidity, wind speed and wind direction produce an increase in the risk of higher pollutant concentrations. Besides, obtained results showed that wind speed and wind direction are the most important variables in the distribution of particles. The results obtained may allow administrations or citizens to support decisions.

## Introduction

The knowledge about how the globe is suffering from continuous atmospheric degradation is what motivates this study which is focused on air pollution in port-cites. Universally, several summits have been developed for decades to tackle shipping emissions. Since until 31st December 2019, for ships operating outside the ECA,^1^ the limit for sulphides content of ships’ fuel oil was 3.50% m/m (mass by mass); from 1st January 2020 onwards, 0.50% m/m limitation must be applied concerning the IMO^2^ established in the 16th October 2008 committee. This deadline was set in the MARPOL treaty. Mainly, air pollution caused by vessels and aviation are referred to sulphur dioxide (SO_2_), nitrogen dioxide (NO_2_) (Rivera et al. 2015) and particle matters (PM) (Chaloulakou et al. 2005; Agrawal et al. 2008; Grivas et al. 2018); thereby, this study is focused on these pollutants. Moored ships are also responsible for smoke in the air in port-cities, basically, due to the powerful engines usage in secondary electricity supply devices (Adamo et al. 2014). Moreover, European Directive 2008/50/EC (EU directive, 2008) establishes several thresholds and an AQI^3^ for every pollutant as mentioned in the study about particular matter with an aerodynamic diameter < 10 µm (PM_10_) (Vicente et al. 2012).

As stated in (Westmoreland et al. 2007), nitric oxide (NO) together with NO_2_ is known as NO_x_ and all kind of high-temperature combustions, as vehicles engines, are related to them mainly in urban areas (Chaloulakou et al. 2008). According to (Carslaw et al. 2007), NO_2_ emissions are more associated with diesel engines. The extent of this research is to assess how is the real air quality scope in a controversial zone to face future predictive studies (Turias et al. 2008; Munoz et al, 2014; González-Enrique et al. 2019a). To tackle researches about air quality, a complex scenario where several pollution sources interact was chosen. The Bay of Algeciras is a strategic point with several oil refineries, steel factories, and other industries. Algeciras port together with Gibraltar airport, which are connected by many roads with plenty of freight transport and constant private traffic, contributes to very complicated air pollution conditions. The second most populated city in the Bay is La Línea with 63,147 inhabitants in 2019. Algeciras is the major town in the Bay with a population of 121,957 inhabitants in 2019. Its port is of real importance not only in Spain but also in the world.

Overwhelming pieces of evidence show that particle pollution in the outdoor air we breathe, those coming from vehicles exhaust pipes (Crabbe et al. 1999; Carslaw et al. 2007; Bozkurt et al. 2018), coal-fired power plants, petroleum refineries and other industrial sources, can cause lung cancer and higher mortality rates in urban areas (Finkelstein et al. 2003). It was observed that several substances, including PM_10_, reached higher levels in urban sites in the winter season (Bozkurt et al. 2018). Long-term pollution exposure to nitrogen oxides or sulphides can contribute to ailments such as cancer or asthma (Clench-Aas et al. 2000; Finkelstein et al. 2003). It is essential to control immissions since these affect human beings. Air pollutants are spread to different cities close to the emission points by winds (Cheung et al. 2020). Previous studies of SO_2_ demonstrated that short-term were better than medium-term predictions, and the reverse in the case of PM_10_ concentrations (Turias et al. 2008). A recent study related to PM pollutants shows that even though when the heavy industries have decreased their manufacturing in the first lockdown period during the SARS-COV-2 (Covid-19) health crisis, severe pollution is not reduced when meteorology is adverse (Wang et al. 2020). Furthermore, it must be considered that if ozone (O_3_) suffers from ozonolysis in presence of high levels of SO_2_ and H_2_O, the potential formation of secondary aerosols depends on relative humidity and meteorological conditions (Diaz-de Mera et al. 2017). Also, secondary aerosols (NO_2_) are formed in the chemical reaction between the NO and O_3_ (Westmoreland et al. 2007). Thus, air pollution in urban centres is a complex toxic-components mixture affected with the weather conditions and with a high impact on inhabitants, above all in those with cardiac insufficiency and respiratory distress (Kolehmainen et al. 2001). PM_10_ pollutant enters the body exclusively through the respiratory system (Vicente et al. 2012). Therefore, it is primordial to identify the temporal evolution of pollutant concentrations in the air in urban regions to ensure the living standard. Immission data were collected from a monitoring network located in this study area by the Environmental Agency of Andalusian Government in the south of Spain. The study region has also a peculiar local meteorological scenario due to the closeness to The Strait of Gibraltar. The study contains descriptive statistical methods and more sophisticated statistical tools, such as *p-values* in regression analysis, or trend predictions.

Citizens or administrations have the need for reliable information about the possible risks they are exposed. One of the main aims of this study: knowing the most important variables and causes of high levels of pollution and, on the contrary, the variables that foster low levels of pollution. In this work, these challenges are achieved through a data-driven approach. Using historical data, a statistical analysis has been performed including three stages: i) descriptive, ii) predictive, and iii) prescriptive, which will be explained more deeply in the next sections.

The rest of this paper is organized as follows. Section 2 describes the data and the case study. Section 3 introduces the methodology. Section 4 presents and discusses the results, and finally, Sect. 5 establishes the main conclusions.

## Materials and Methods

**Fig. 1:**
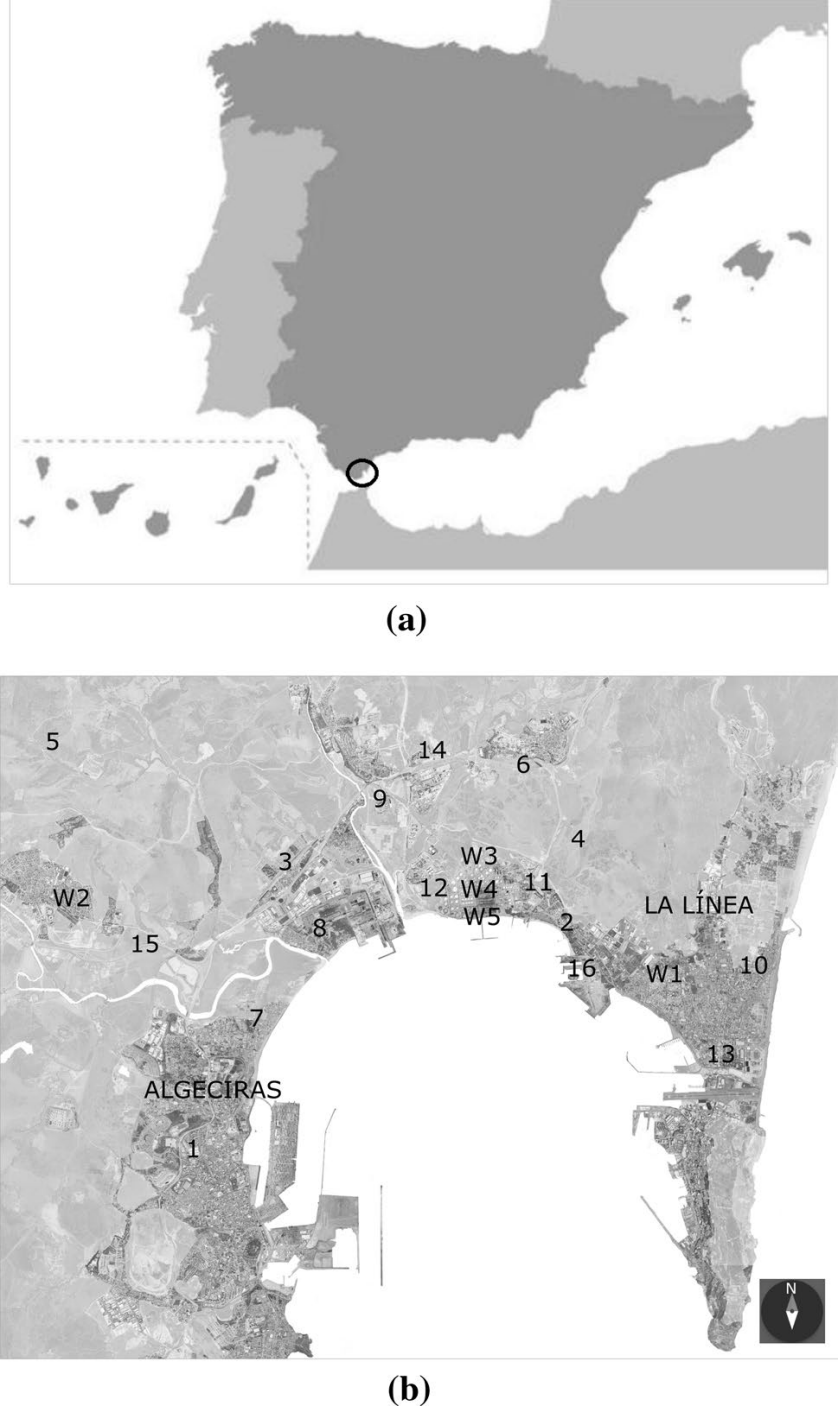
Area of study with the monitoring stations: **a** Site location: Bay of Algeciras (South of Spain) and **b** Location of pollutants and meteorological monitoring stations, **b** (Scale = 1/100000). The monitoring stations codes are collected in Tables 1 and 2

This section introduces a description of the database. A map of the area of study with reference to Spain is shown in Fig. 1(a), and a general location of the Bay of Algeciras is shown in Fig. 1(b). In this figure, the position of Algeciras port can be seen with its massive dimension, a total length of 17,750^4^ m amongst berths and seawalls. The situation of the two main cities of the bay, where this study focuses, Algeciras and La Línea, are located in front of each other as seen in Fig. 1(b). There are two dominant winds in this region, Levante (East) and Poniente (West), which seem to be produced by the situation of the bay in the proximity of the Strait of Gibraltar in connection with the Mediterranean Sea and the Atlantic Ocean. This special location of the bay creates a powerful air stream. Besides, as shown in Fig. 1(b), the pollutants and meteorological stations are spread over the bay.

Naturally, a higher amount of available resources in data collection, such as sampling points and sensors, will chaise more reliable management of immission measuring. The Andalusian Government maintains its air quality monitoring station system throughout the Bay, where pollutant concentrations and atmospheric parameters were collected from the period of 2010 to 2015 and kindly provided to the University of Cádiz. The Bay of Algeciras counts on sixteen monitoring stations for collecting air pollutants and five meteorological specialized sensors. Three of the weather sensors are located inside a petroleum refinery (CEPSA) at three different heights (Table 1). Table 2 shows every pollutant in the monitoring stations. NO_2_ pollutant is recorded in fourteen stations, SO_2_ in sixteen stations, and PM_10_ in ten stations.

**Table 1:** Meteorological monitoring stations

Code	Weather station description
W1	La Línea
W2	Los Barrios
W3	Cepsa 10 m high
W4	Cepsa 60 m high
W5	Cepsa 15 m high

**Table 2:** Monitoring stations and the pollutants measured. NO_2_ is recorded in a total of fourteen stations, SO_2_ in sixteen, and PM_10_ in ten stations

Code	Description of stations	Collected pollutants
1	Algeciras (EPSA)	NO_2_, SO_2_, PM_10_
2	Campamento	NO_2_, SO_2_
3	Los Cortijillos	NO_2_, SO_2_, PM_10_
4	Hostelería	NO_2_, SO_2_
5	Alcornocales	SO_2_, PM_10_
6	Carteya	NO_2_, SO_2_, PM_10_
7	Rinconcillo	NO_2_, SO_2_, PM_10_
8	Palmones	NO_2_, SO_2_, PM_10_
9	San Roque	NO_2_, SO_2_, PM_10_
10	El Zabal	NO_2_, SO_2_, PM_10_
11	Economato	NO_2_, SO_2_
12	Guarranque	NO_2_, SO_2_
13	La Línea	NO_2_, SO_2_, PM_10_
14	Madrevieja	NO_2_, SO_2_
15	Los Barrios	NO_2_, SO_2_, PM_10_
16	Puente Mayorga	SO_2_

The data have been collected hourly during the period of six years, from 2010 to 2015, with an apparent total database of 52,560 hourly data. Besides, twenty-four meteorological variables, described in Table 3, were collected hourly, as well. Following previous works (Turias et al. 2008; Munoz et al, 2014; González-Enrique et al. 2019b, 2019c), a procedure of missing data imputation was developed as a preprocessing step. The meteorological variables analysed are wind speed (WS), wind direction (WD), solar radiation (SR), atmospheric pressure (AP), rainfall (RF), relative humidity (RH), temperature (T) and the pollutants are NO_2_, SO_2_ and PM_10_. These are the main substances expelled by the principal sources of air pollution in this area.

**Table 3:** Meteorological variables

Variable	Description
W1:WD	Wind direction (degrees)
W1:RH	Relative humidity (%)
W1:RF	Rainfall (l/m2)
W1:Tª	Temperature (°C)
W1:WS	Wind speed (km/h)
W2:WD	Wind direction (degrees)
W2:RH	Relative humidity (%)
W2:RF	Rainfall (l/m2)
W2:AP	Atmospheric pressure (hPa)
W2:SR	Solar radiation (w/m2)
W3:RH	Relative humidity (%)
W3:RF	Rainfall (l/m2)
W3:AP	Atmospheric pressure (hPa)
W3:SR	Solar radiation (w/m2)
W4:WD	Wind direction (degrees)
W4:Tª	Temperature (°C)
W4:WS	Wind speed (km/h)
W5:WD	Wind direction (degrees)
W5:WS	Wind speed (km/h)
W5:RH	Relative humidity (%)
W5:RF	Rainfall (l/m2)
W5:AP	Atmospheric pressure (hPa)
W5:SR	Solar radiation (w/m2)
W5:WS	Wind speed (km/h)

After data imputation, this study analyses the concentrations database together with the weather variables. Statistical parameters are obtained in every station to study their correlations and to study trend connections with other stations (descriptive approach). Then multivariate regression models (Romero et al. 2020) have been established for every pollutant in each station to check the relevant features and to dispose of estimation models to chaise a virtual sensor with numerous applications, such as, missing data imputation, a real-time usage in a robust monitoring net or prediction of the air pollution (a predictive approach). Previously, authors have used these methods (Turias et al. 2008; Munoz et al, 2014; Moscoso-López et al. 2019; Ruiz-Aguilar et al. 2021) in different works. Finally, measuring higher values of immission pollutants concentrations, a complete assessment of relative risks was developed to have at our disposal cause-effect knowledge about which are the main hazardous variables to prevent and take decisions (a prescriptive approach).

## Descriptive analysis

### Correlation

**Fig. 2:**
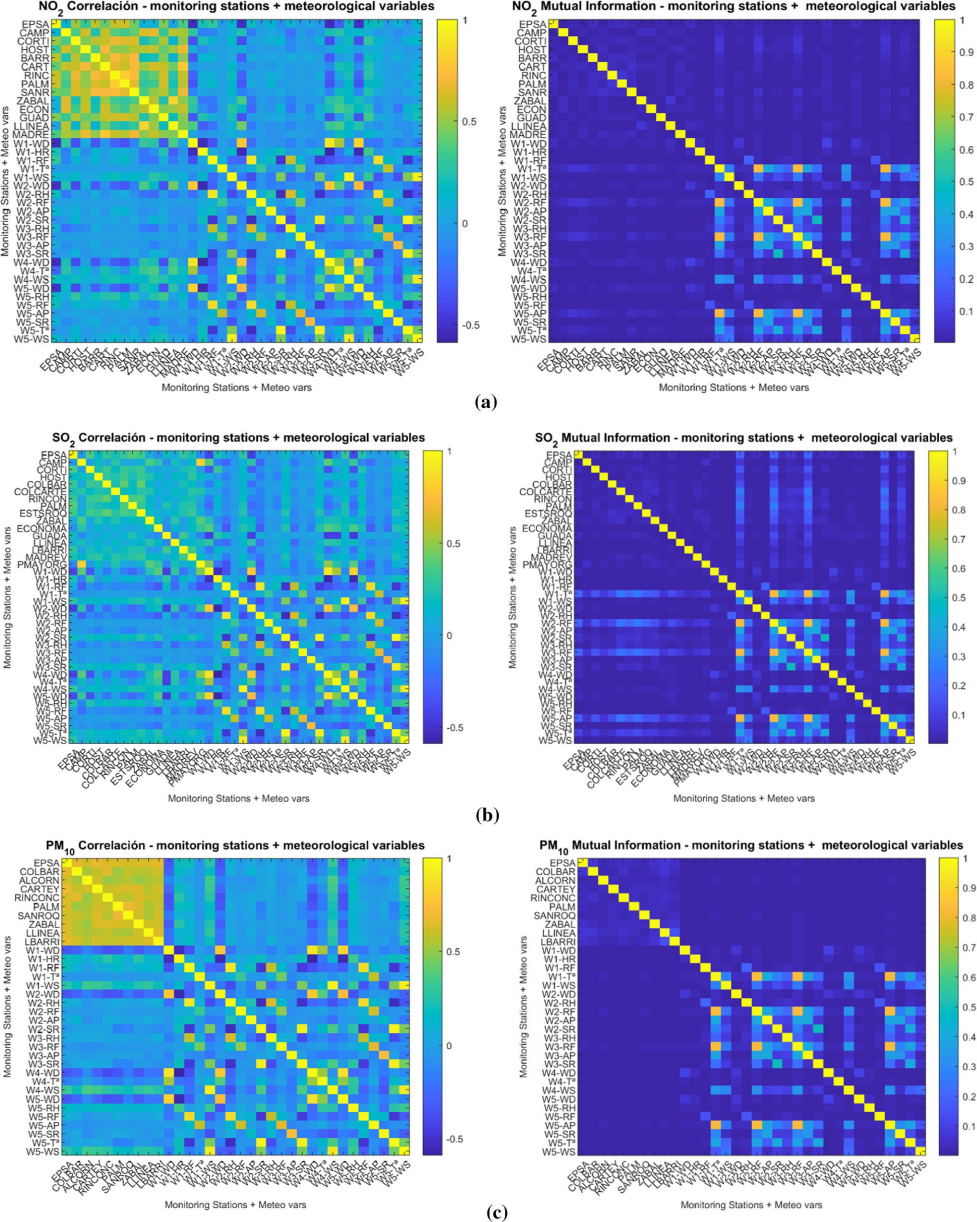
Plots of correlation values and mutual information among pollutants monitoring stations and meteorological variables **a** N0_2_, **b** SO_2_, **c** PM_10_

An in-depth statistical assessment was developed to get a general idea of the scope of the pollution in the study area. In Table 4, the mean, median, mode, variance, kurtosis and skewness are collected for every pollutant and study city. On the other hand, a linear correlation analysis was developed. A correlation is a reciprocal relation between two or amongst different variables which are expected to have some kind of connection, even though correlation does not mean relation. For instance, if one of these variables grows, the other is expected to increase or even decrease. Multidimensional correlation results were computed (see Fig. 2a, b and c). Pearson correlation coefficient (*r*) is shown in Eq. (1) where the numerator corresponds to *COV(X,Y)*, the covariance between every pair of independent variables, and the denominators are, respectively, σ_x_ · σ_y_, the typical deviations of them.

1 $$r = \frac{{\mathop \sum \nolimits_{i = 1}^{n} \left( {xi - \vec{x}i } \right)\left( {yi - \overline{y}i} \right)}}{{ \sqrt {\mathop \sum \nolimits_{i = 1}^{n} \left( {xi - \overline{x}i } \right)^{2} } \sqrt {\mathop \sum \nolimits_{i = 1}^{n} \left( {yi - \overline{y}i} \right)^{2} } }}$$

**Table 4:** Descriptive statistical measurements in Algeciras (1) and La Línea (13) monitoring stations

Station	Mean (µg/m^3^)	Median (µg/m^3^)	*σ*^2^	Kurtosis	Skewness
*NO*_*2*_					
1	28.850	25.000	460.680	3.587	0.910
13	26.189	19.833	408.510	4.669	1.331
*SO*_*2*_					
1	11.000	8.333	67.818	39.726	3.867
13	11.966	10.000	64.693	19.789	2.813
*PM*_*10*_					
1	27.409	24.543	295.620	25.456	2.803
13	30.745	27.333	405.500	67.438	5.140

### Mutual information

Mutual information (MI) (Shannon, 1948) is based on Shannon’s information theory. MI measures the statistical dependence between two variables, and thus, independency implies a low mutual information between them. Mutual information is a nonnegative measure (Kullback, 1968). MI uses the concept of entropy as a measure of uncertainty, since its maximum is when all values have equal probability of occurrence (Shannon, 1948). The advantage of mutual information is its ability to estimate a general dependence between variables, as opposed to correlation between variables where only linear relationships are considered.

To specify how much common certainty there are in two data samples, MI uses the concept of crossentropy $H\left( {X,Y} \right)$:

2 $${\text{MI}}\left( {X,Y} \right) = H\left( X \right) + H\left( Y \right) - H\left( {X,Y} \right)$$

where $H\left( X \right)$ is the entropy of $x$ variables and $H\left( Y \right)$ is the entropy of the output $y$ (Cover and Thomas, 1993).

MI can be computed using Eq. 2 through an estimation adaptive partitioning (Darbellay and Vajda, 1999) procedure as authors previously used in González-Enrique et al. (2021). The ITE Toolbox (Szabó, 2014) has been applied in this work for MI calculation.

### Trend analysis

In order to study trends between the monitoring stations for each air pollutant, we have used nonparametric trials and non-normality tests such as Wilcoxon rank test, Wilcoxon signed test and Wilcoxon signed-rank test to be compared with T-test (Box et al. 1976). Data are grouped into pairs of observations or monitoring stations, ($x$_i,_$y$_i_), which are aimed at getting to know if every pair is equal or not from a statistic point of view. The assumptions in these models are that if $y$_i− _$x$_i_ ≠ 0, samples are independent, and they have a continuous and symmetric distribution concerning the same common median $\theta$. These tests consist in returning a logical value indicating the test decision within a confidence interval, conventionally, of 95%. The decision will vary according to the kind of test we use. Basically, if the logical value it returns is 1, this indicates a rejection of the null hypothesis, and if the logical value it returns is 0, this indicates a failure to reject the null hypothesis at 5% of the significance level. *Wilcoxon rank test* is used when two samples have different lengths and they are independent. It tests the null hypothesis that data are samples from continuous distributions with equal medians, against the alternative that they are not. *Wilcoxon sign test* is the simplest nonparametric test applied in paired samples (Wasserman, L., 2004a). It is usually used to test the median of a population and returns the *p-value* for a two-sided sign test through a binomial distribution (Wasserman, L., 2004b). *Wilcoxon signed-rank test* is used instead of a T-test when normality in the sample cannot be proved, besides this test is the nonparametric of the dependent samples *T test*. A T test is used to compare two groups determining if there is a significant difference between their means, which might be related to certain features. Results of the several tests applied are collected in Table 5.

**Table 5:** Trend tests for similarity amongst monitoring stations

*Daily data test*			
Test	NO_2_	SO_2_	PM_10_
Wilcoxon rank	1,13	1,13,15	1,10,15; 7,8,13
Wilcoxon signed	–	1,13,15	1,15; 7,10,13
Wilcoxon signed-rank	–	1, 15	7,10,13
T test	–	1, 15	1,15; 7,8,10,13
*Weekly data test*			
Wilcoxon Rank	–	1, 15	7, 10, 13
Wilcoxon Sing	–	1, 15	–
Wilcoxon singed-rank	–	1, 15	10,13
T test	–	1, 15	7,10,13

### Predictive analysis

Linear regression is a linear approach to modelling the relationship between two variables, used as a predictive tool for studying the response of one dependent variable, or response $Y$, and the covariate variable $X$, also called feature or predictor (Eq. 3).

3 $$R\left( x \right) = E\left( {{\raise0.7ex\hbox{$Y$} \!\mathord{\left/ {\vphantom {Y X}}\right.\kern-\nulldelimiterspace} \!\lower0.7ex\hbox{$X$}} = x} \right) = \int {yf\left( {{\raise0.7ex\hbox{$y$} \!\mathord{\left/ {\vphantom {y x}}\right.\kern-\nulldelimiterspace} \!\lower0.7ex\hbox{$x$}}} \right)} dy$$

Multiple regression is a *many-to-one* relationship amongst independent variables $x$_i_ and the response $y$. Adding more predictors to the model does not mean a better response, and it may produce *overfitting* or *multicollinearity*. The sum of linear parameters and an error gives the multiple regression model (4).

4 $$yi = \mathop \sum \limits_{i = 1}^{n} (\beta o + \beta i\cdot Xij ) + \varepsilon {\text{i}}$$

where $\beta {\text{o}}$ is the intercept, $\beta i$ are coefficients which show the weight of every independent variable $Xi$, and Ɛ${\text{i}}$^5^ is the error or residual. When this equation is estimated, we get the predicted value of the dependent variable, $\hat{y}i$, obtaining $b0, b1, b2,...bi,$ the estimates of $\beta {\text{o}}$, $\beta 1$, $\beta 2$,…,$\beta {\text{i}}$, respectively.

As it is well known, the coefficient of determination, R^2^, is preferably closer to one as possible. In addition, the *p-value* of each variable was calculated in order to discard all of the redundant independent variables whose *p-value* is ≥ 0.01. Generalization is key in data analysis since it is the ability to get good results with new unseen data. Building a model that fits the data sorely well does not always guarantee that the model is useful. The model should work well not only with the data it has learned from but also with the data that have not been used so far. The procedure to measure generalization is to divide our data into two sets: the training set and the test set. The training set, larger than the test set, contains the data used by the model to learn the parameters, and the test set contains unseen data not used in learning. The test set will be used to know how the model behaves with new data. In this analysis, the period 2010–2014 is used as a training set and the year 2015 is used as the test set. The results have been collected to these “new” data.

### Prescriptive analysis

An analysis can be descriptive or predictive when representing an event, or prescriptive when researching a cause-effect occurrence, and this fact can be used in order to have a support decision tool (Schmidt and Kohlmann, 2008). The consequence can be evaluated by the relative risk analysis, which is explained briefly here. There are two methods for measuring the risk: *Odd Ratio* and *Risk Ratio*, both dimensionless and accompanied by the confidence interval (CI), which is a measure of the precision of the estimation (Tripepi et al. 2007). Their usage depends on the design of our study.

*Odds Ratio (OR)* it is used when a retrospective design is applied. This means that the first step is focusing on the consequences and then analysing the causes. The odds are a way of representing probability (Tripepi et al. 2007). OR is also known as “Cases and Controls'' (Ganguly 2006). Basically, odds is the ratio that represents the probability of occurrence of an event by means of the quotient between the happening event probability and the non-happening event probability (Bland and Altman 2000). It indicates how higher is the probability of occurrence of an event towards its non-occurrence (Sumargo 2018). These terms are described below.

5 $${\text{OR}} = \frac{{{\text{Odds}}1}}{{{\text{Odds}}0}} = \frac{{{\raise0.7ex\hbox{${R_{1} }$} \!\mathord{\left/ {\vphantom {{R_{1} } {\left( {1 - R_{1} } \right)}}}\right.\kern-\nulldelimiterspace} \!\lower0.7ex\hbox{${\left( {1 - R_{1} } \right)}$}}}}{{{\raise0.7ex\hbox{${R_{0} }$} \!\mathord{\left/ {\vphantom {{R_{0} } {\left( {1 - R_{0} } \right)}}}\right.\kern-\nulldelimiterspace} \!\lower0.7ex\hbox{${\left( {1 - R_{0} } \right)}$}}}} = \frac{{a_{1} b_{0} }}{{a_{o} b_{1} }}$$

*Risk Ratio (RR)* also called relative risk. It is a statistical concept used as a measure of association between dependent and independent variables. It is indicated to prospective studies, beginning in the reviews of the causes and their supervision until examining the consequences. RR is also called “Cohort study'' (Finkelstein et al. 2003; Schechtman 2002). The relative risk can be calculated as the ratio between the two incidence proportions or two incidence rates (Tripepi et al. 2007). It is the quotient between the proportion of cases with risk factor (subindex 1) and the proportion of cases without risk factor (subindex 0).

6 $${\text{RR}} = \frac{R1}{{R0}} = \frac{{{\raise0.7ex\hbox{${a_{1} }$} \!\mathord{\left/ {\vphantom {{a_{1} } {n_{1} }}}\right.\kern-\nulldelimiterspace} \!\lower0.7ex\hbox{${n_{1} }$}}}}{{{\raise0.7ex\hbox{${a_{0} }$} \!\mathord{\left/ {\vphantom {{a_{0} } {n_{0} }}}\right.\kern-\nulldelimiterspace} \!\lower0.7ex\hbox{${n_{0} }$}}}}$$

The terms are calculated counting and separating the individuals of the sample in cases-no factor, cases-factor, no cases-no factor, and no cases-factor. The total of individuals with no factor is *n*_*0*_ and the total of individuals with factor is *n*_*1*_. The sum of individuals with cases-no factor is *a*_*0*_, the sum of individuals with cases-factor is *a*_*1*_, the sum of individuals with no cases-no factor is *b*_*0*_, and the sum of individuals with no cases-factor is *b*_*1*_. Thus, it is easy to imagine that *n*_*0*_ = *a*_*0*_ + *b*_*0*_ and *n*_*1*_ = *a*_*1*_ + *b*_*1*_.

For instance, the relative risk of a situation is the ratio of risks of the treated group and the control group (Schechtman 2002). Observing Eqs. (5) and (6) is immediate to realise that when *OR* > *1* or *RR* > *1*, a positive association, the presence of the factor is related to a higher occurrence of the event, and the reverse if the association is negative, *OR* < *1* or *RR* < *1* (Finkelstein et al. 2003). Conversely, when *OR* = *1* or *RR* = *1*, there is no association between the presence of the factor and the event. There are several existing linkages between OR and RR. RR is more perceptive. OR lets us adjust by confounding variables through logistic regression, although it is not applied in this study.

## Results and discussion

An overview of the in-depth analysis is shown in this section. Firstly, a descriptive statistical assessment was performed showing the most relevant features. Likewise, a linear correlation analysis was conducted to find out and determine which pairs of variables shared information. Also, a trend analysis was developed in order to establish those stations with similarities. Secondly, a linear multiple regression analysis was performed allowing to determine which variables are the most explanatory for every pollutant and location (with *p-value* < 0.01), and besides, to be able to establish how much variability can be fully explained with these models. These regression models can serve as virtual sensors because they are capable of inferring a measure (the concentration of a pollutant in a certain location) based on other variables. Therefore, they can be used for the imputation of missing data or as robust control in a monitoring network. Similarly, or with the same objectives, an approximation has been made to calculate which monitoring stations are equivalent to others, in this case using statistical comparison tests on their means.

UNECE^6^ announced that air pollution is now considered to be the world’s largest environmental health threat, accounting for 7 million deaths around the world every year. The main substances affecting health are: nitrogen oxides (NO_x_), sulphur oxides (SO_x_), ozone and particulate matter. Both extent and duration of the exposure influence health diseases. This study and its results are interesting in order to give information about air pollution to citizens. Besides, regarding the economic costs of air pollution, WHO^7^ and OECD^8^ estimated in 2015 that the amount of money related to premature deaths and disabilities in Europe reached almost USD 1.6 trillion. Therefore, preventing long exposures could be useful to avoid risk factors for major diseases.

Finally, some results are collected on the statistical risks of interactions of the variables. Thus, we have obtained the most influenced interaction values for pollutants (especially in the case of the highest pollution values or, in contrast, of the lowest).

### Descriptive analysis

The statistical terms of mean, median, variance, kurtosis, and skewness are collected in Table 4 for each pollutant recorded in the stations in both cities. Simply looking at this board we can have an idea of their relevance. Generally, the means of pollutant concentrations are very similar in Algeciras (number 1) and La Línea stations (number 13). Roughly, during the study period, it was observed that the highest mean occurs in La Línea for all pollutants except for NO_2_. All medians are beneath the mean concentrations which indicate that fifty percent of concentration values are upper the median, proof that the database does not follow a normal distribution. This is also appreciated with a positive skewness (> 0) when symmetry tends to values higher than the mean. The sharpest graph is the one with the highest kurtosis coefficient, corresponding to the PM_10_ in La Línea monitoring station (13).

### Correlation

Figure 2 exposes the correlation values for every pair of variables. These plots represent the resulting correlation results, showing in colours closer to yellow the values of highest correlation coefficients tending to one. Coefficients of one, those of bright yellow in the diagonal, correspond to every variable with itself (it is not representative). Hereafter, the most significant correlation values in Algeciras and La Línea stations with the rest monitoring stations and atmospheric variables are exposed here. Speaking in absolute values, the correlations do not exceed *r* = 0.6781, which correspond to Carteya station correlated with Algeciras station for the PM_10_ pollutant and, continuedly, also the stations Los Barrios and El Zabal show similar correlation. For this pollutant, similar values of correlations are depicted in La Línea for Los Barrios, Algeciras, and El Zabal stations. Observing weather variables for PM_10_, wind direction is the highest correlated variable in Algeciras station and wind speed in La Línea. Moreover, regarding weather variables, the highest values of correlation connect wind direction in weather stations W1, W2, and W4 with Algeciras station for NO_2_. Besides, this pollutant in Algeciras station presents a correlation with Palmones, El Rinconcillo, and Los Barrios stations. On the other hand, La Línea station presents a connection with Campamento, Escuela de Hostelería, and Los Barrios stations for the same pollutant. In the case of La Línea station for the NO_2_ pollutant, the weather variables more correlated are wind direction in W1, W2 stations, and temperature in W4. Finally, for SO_2_ wind direction is the weather variable that most correlates both cities and also relative humidity in La Línea station measured in W3. Considering monitoring stations for SO_2_, Algeciras is correlated with Palmones, Los Cortijillos, and San Roque stations. La Línea is correlated with El Zabal, Puente Mayorga, and Campamento stations, which makes sense due to their proximity.

### Mutual information

In order to get knowledge about the nonlinear behaviour amongst the variables, MI has been computed. In this sense, MI provides a different and more general criterion for investigating relationships between variables.

Regarding to the MI results between the studied variables, it is observed that there is not much nonlinear information between the monitoring stations. The maxima were found in the combinations of the meteorological variables W1-T, W2-RF, W3-RF, and W5-AP. This information complements to that calculated with the linear correlation and allows us to assume that these variables could be used in nonlinear regression or prediction models. Nevertheless, most of the shared information is linear rather than nonlinear with the limits of values observed in Fig. 2 (maximum 0.7 approximately). The behaviour is very similar for the three studied pollutants, with higher MI values only in the case of SO_2_ in the monitoring stations when a combination with the variables W1-T, W2-RF, W3-RF, and W5-AP was tested.

### Trend analysis

**Fig. 3:**
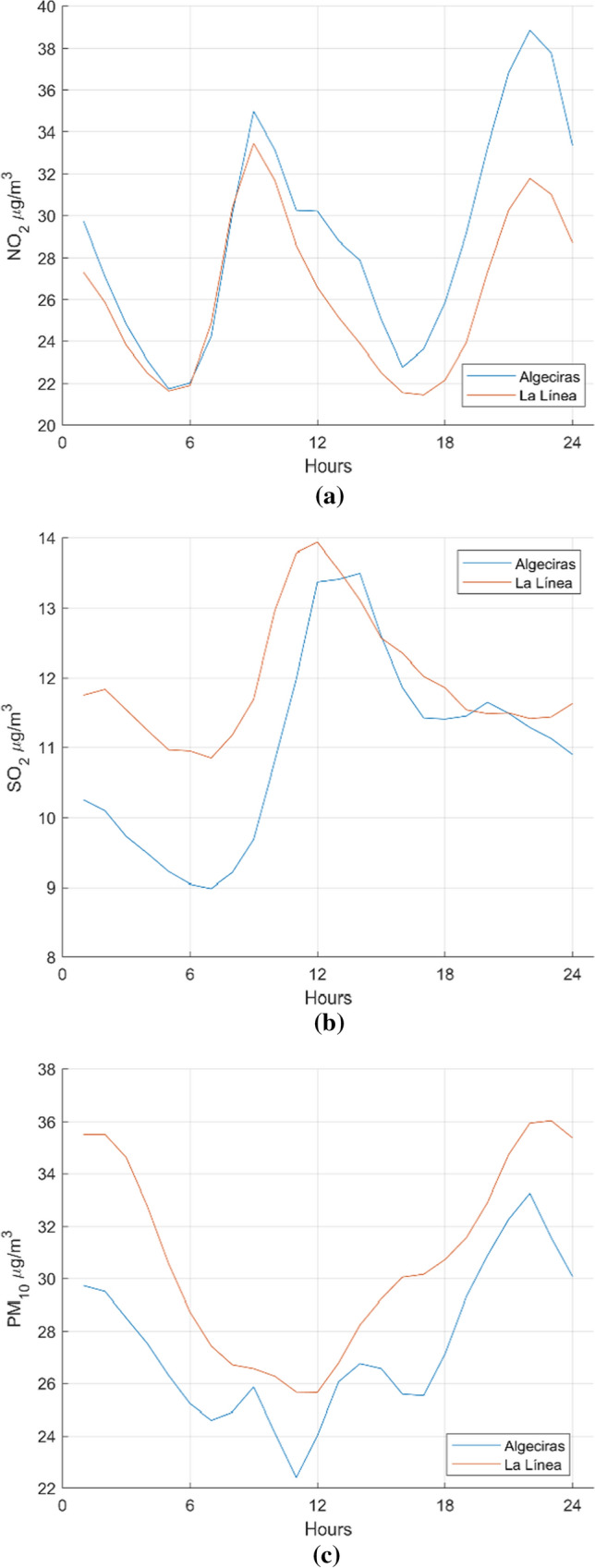
Average day. Comparison between the hourly mean concentration of pollutants in Algeciras and La Línea during the period 2010–2015: **a** NO_2_ **b** SO_2_ **c** PM_10_

**Fig. 4:**
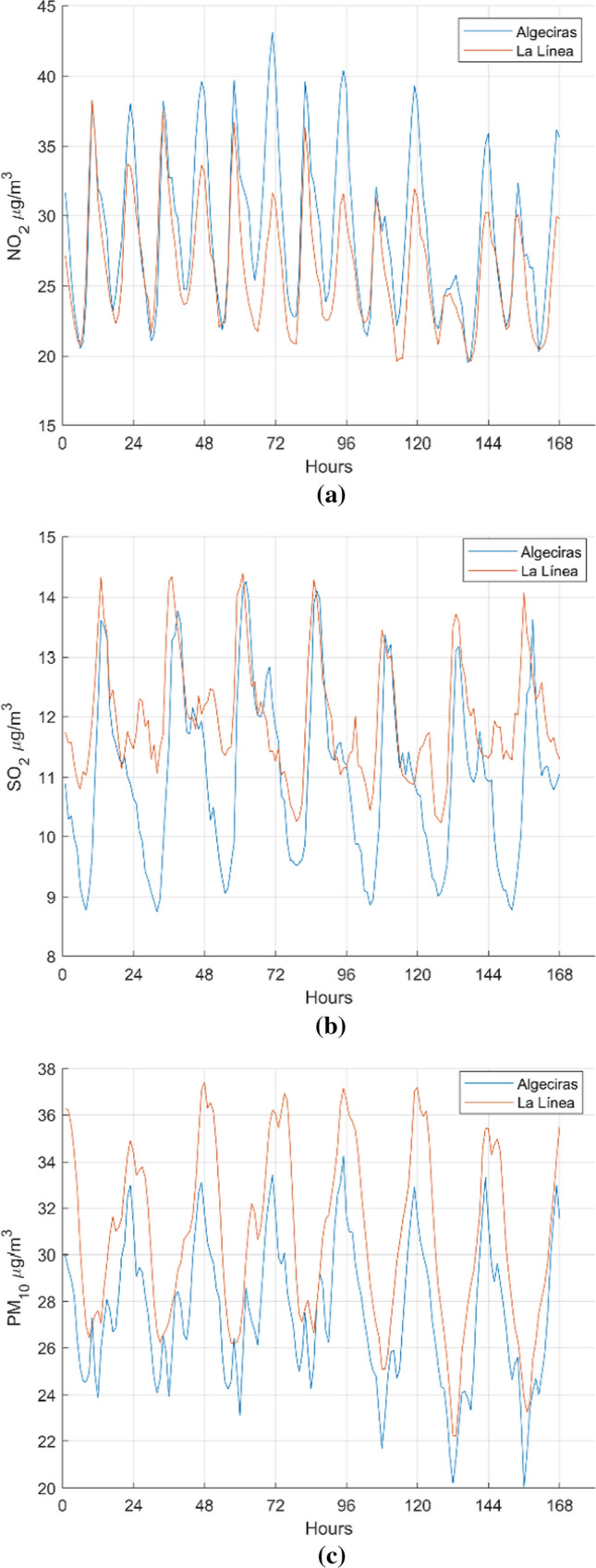
Average week. Comparison between the hourly mean concentrations of pollutants in Algeciras and La Línea during the period 2010–2015: **a** NO_2_ **b** SO_2_ **c** PM_10_

The tendency is a measurement of variability between two samples regarding to their means. In this section, two samples coming from different stations are measured in order to obtain if they have the same trend or not from a statistical point of view, using, henceforth, hypotheses test for both daily and weekly means. In daily calculation, all database is divided into twenty-four intervals, or hours, and the mean is calculated for every hour (see Fig. 3). In weekly calculation, data are divided into groups of 52 weeks in order to calculate the mean of every interval that is the weekly mean for the total of the six years (see Fig. 4). The figures show daily data that give the hourly mean concentration for every pollutant in Algeciras (EPSA) and La Línea (Fig. 3), and weekly data giving the daily mean concentration (Fig. 4). In these figures, we can see that the highest levels of SO_2_ and PM_10_ pollutants mainly affect the city of La Línea, and for the NO_2_ pollutant, the highest level is obtained in Algeciras. Checking Fig. 3(a), a NO_2_ concentration level peaks appears at 38.88 µg/m^3^ which corresponds to the city of Algeciras in contrast to the highest concentration level peak of 31.79 µg/m^3^ in La Línea. In both cities, the NO_2_ concentrations grow strikingly at 10 p.m., together with a lower peak at 10 a.m. In Fig. 4(a) is noticed that the highest level of NO_2_, 43.17 µg/m^3^, corresponds to Algeciras achieved on Wednesday, and in La Línea the highest level of NO_2_ is 38.29 µg/m^3^ obtained on Monday. In the case of SO_2_, Fig. 3(b) shows in La Línea a steadily rise until the highest value of 13.94 µg/m^3^ at 12 a.m. and very close, 13.49 µg/m^3^ at 2 p.m. in Algeciras. Figure 4(b) shows that Wednesday is the day of highest values, very similar both in La Línea (14.39 µg/m^3^) and Algeciras (14.26 µg/m^3^). Concerning PM_10_ pollutant, Fig. 3(c) depicts a steep downward in both cities at 12 p.m. and also the highest mean-hour is 36.04 µg/m^3^ corresponding to La Línea at 11 p.m. In Algeciras, the value is 33.28 µg/m^3^ at 10 p.m. Figure 4(c) shows the highest value of 37.41 µg/m^3^ in La Línea corresponds to Tuesday and in Algeciras 34.27 µg/m^3^ the Thursday. In general, at Algeciras station exists a major fluctuation for every pollutant, obtaining the lowest values of concentrations. At La Línea station, the values are maintained higher, probably caused by its proximity with industrial environment combined to west winds. On the other hand, after applying several trend-tests for both daily and weekly data to get those stations with similar behaviour, promising trend results were obtained for NO_2_, SO_2_ and PM_10_ in the cities of Algeciras and La Línea (see Table 5). Trend results for similar stations demonstrate that for SO_2_ pollutant Algeciras station (number 1) is always similar to Los Barrios station and in the case of Wilcoxon rank and Sign tests is also equal to La Línea station (number 13). This argument is used in the rest of tests. Regarding PM_10_, the situation is strongly different since several stations show similar behaviour with all tests except Wilcoxon sign for weekly data test, which presents none of them.

### Predictive analysis

A Multiple linear regression (MLR) analysis was applied in order to estimate the three different air pollutants as a function of meteorological variables and the rest of monitoring stations. In this work, the estimation of the parameters was done using the data in the period 2010–2014 (training or design set) and the regression results were collected using the year 2015 (as a test set). The best regression model is obtained when highest R^2^ and lowest MSE. Regression results in Algeciras and La Línea are displayed in Table 6. We observe that in Algeciras station the best regression value is for PM_10_ and in La Línea for NO_2_, nevertheless, SO_2_ presents similar values in both cities. Finally, using the *p-value* < 0.01 of the regression, the most relevant features can be selected in every model. Their weights or estimates ($\beta i$) and the intercept ($\beta 0$) of each regression equation are shown in Table 7 along with the most relevant variables for every pollutant and station. Those variables with positive estimates indicate that the dependent variable (i.e. each air pollutant concentration) are positively affected by these variables and reversely with negative sign. For instance, regarding the weather variables, Algeciras station is positively influenced by wind speed in W4 and negatively in W1 for all pollutants. Considering the regression among monitoring stations, Algeciras is more positively affected by Los Barrios for all pollutants and also by Palmones station in the case of SO_2_. Besides, Algeciras is affected negatively by San Roque for NO_2_. In the case of La Línea station, the variables that more influence positively are wind speed (in W1 and W4 weather variables) for all pollutants and also wind direction (in W1 for SO_2_). Regarding monitoring stations, La Línea is positively affected by El Zabal in the case of NO_2_ and SO_2_ pollutants and Los Barrios station in the case of PM_10_.

**Table 6:** Multiple linear regression values for every pollutant in Algeciras (1) and La Línea (13) monitoring stations

Pollutants	Monitoring stations	R^2^(MLR)	MSE(MLR)
NO_2_	1	0.7360	269.14
	13	0.8655	150.60
SO_2_	1	0.6382	33.82
	13	0.5346	30.03
PM_10_	1	0.8244	96.92
	13	0.6608	221.09

**Table 7:** Relevant weather variables and monitoring stations (with regression *p-values* < 0.01) are shown for each pollutant in monitoring stations of Algeciras (1) and La Línea (13). *β*_*i*_ are the estimates, and *β*_*0*_ is the intercept of the regression models

Pollutants	Stations	Relevant weather variables	$\beta i$	Relevant stations	$\beta i$	$\beta 0$
NO_2_	1	W4:WS	0.6363	15	0.3207	− 146.6
		W5:WS	0.2200	7	0.3225	
		W1:RF	0.2151	9	− 0.2004	
		W1:T	− 0.2429	14	0.1564	
		W1:WS	− 0.4134	10	0.1448	
	13	W1:WS	0.3890	10	0.8554	− 21.678
		W4:WS	0.3036	4	0.0915	
		W1:RH	− 0.3871	14	0.0827	
		W5:WS	− 0.3730	3	− 0.0605	
SO_2_	1	W4:WS	0.4007	8	0.1486	− 72.443
		W1:RF	0.0956	15	0.1022	
		W1:WS	− 0.1457	7	0.0938	
	13	W1:WD	0.1462	10	0.1317	− 64.369
		W4:WS	0.1009	15	0.1309	
		W1:RH	− 0.1441	1	0.1112	
		W5:WD	− 0.1713	3	0.0378	
PM_10_	1	W4:WS	0.6752	15	0.1858	− 76.698
		W2:RH	0.1019	6	0.1530	
		W1:T	− 0.1658	5	0.1241	
		W1:WS	− 0.4414	13	0.0975	
	13	W1:WS	0.9279	15	0.2586	− 25.918
		W1:T	0.2768	1	0.1720	
		W2:RF	− 0.3222	5	0.1489	
		W5:WS	− 0.3846	7	0.1348	

In contrast, La Línea station is negatively affected by Cortijillos station for the NO_2_ pollutant. Negatively, the weather station that more leverages La Línea station is W5, wind speed (in the case of NO_2_ and PM_10_) and wind direction in the case of SO_2_. The rest of the values can be depicted in the same way. Tables 1, 2, 3, and Fig. 1 can be checked to locate every station and to observe these interesting relations.

### Prescriptive analysis

In this study, the relative risks RR > 1.25 have been considered as long as statistically significant with a *p-value* < 0.05 in a *Chi test*. The highest and significant RR values in stations are shown in Table 8. Considering the NO_2_ pollutant measured in W2, lower degrees (first quartile, Q_1_) of wind direction (WD) produce the highest relative risk (3.08) of suffering from an elevation of this pollutant above the mean in Algeciras station. Furthermore, we observe that higher degree values of wind direction (third quartile, Q_3_) in W2 do not present any risk of enduring a rise of NO_2_ in La Línea station above the mean. In fact, they act as a protection in this station for this pollutant and weather variable and also occur to WD recorded in W1. Focusing on the SO_2_ pollutant, if the relative humidity (RH) is high, measured in weather station W1 (this means that it is located in fourth quartile, Q_4_), produces the existence of high relative risk (3.48) to suffer from SO_2_ rise (having a value greatest that the mean) in Algeciras station as also happens to wind speed measured in W5 station. Nevertheless, higher values of temperature and wind direction (Q_4_) recorded in W4 station present protection in La Línea station for SO_2_. In the case of PM_10_, in Algeciras station higher values (Q_4_) of wind speed measured in W5 and W4 stations act as a protection of not suffering from an elevation above the mean concentration. However, in La Línea station higher values (Q_4_) of wind speed, in W4 and W1, do affect the risk increase of undergoing overruns above the mean.

**Table 8:** Highest relative risks computed between pollutants and meteorological variables. Risk 0 means non-risk (protection), risk 1 means risk

Pollutants	Monitoring stations	Meteorological variables	Risk	Quartile	RR
NO_2_	1	W2:WD	1	1	3.08
	13	W2:WD	0	3	2.96
	13	W1:WD	0	3	2.35
SO_2_	1	W1:RH	1	4	3.48
	1	W5:WS	1	4	3.30
	13	W4:T	0	4	2.60
	13	W4:WD	0	4	2.58
PM_10_	1	W5:WS	0	4	2.65
	1	W4:WS	0	4	2.63
	13	W4:WS	1	4	2.31
	13	W1:WS	1	4	2.31

## Conclusion

An exhaustive statistical analysis in a complex scenario characterized by a real industrial region along with a quite singular meteorological situation was performed in this study. In general terms, in the period analysed it is observed that Algeciras station recorded higher values of NO_2_ pollutant than La Línea station, however, considering SO_2_, the values were very similar. In the case of PM_10_, La Línea station collected the highest values.

Regarding regression models, the hypothesis that winds are important in this area is tested in this study, showing the relevance of correlation and highest regression coefficients for the two wind components (speed and direction) measured in different weather stations amongst other variables. Regression rates go from values of R^2^ above 0.5346 to the max R^2^ of 0.8655 that corresponds to NO_2_ with La Línea station. Moreover, wind speed appears to be the most relevant variable in the majority of cases in both cities. The two wind components trigger an apparent particle movement which leads to an air-cleaning effect in The Bay of Algeciras. However, Algeciras is highly affected by east winds (Levante) and La Línea is more impacted by west winds (Poniente). Generally, considering the monitoring stations, Los Barrios station presents higher affection with Algeciras for all pollutants. In La Línea, El Zabal station seems to be more relevant. Both of them could be used with relevance in a robust virtual sensor of Algeciras and La Línea stations along with wind variables.

Considering relative risk results, Algeciras is affected negatively by lower degrees of wind direction for the NO_2_ pollutant. For SO_2_ the variables that affect the most to Algeciras are higher values of relative humidity, which produce a rise of SO_2_ above the mean. This increase might be in concordance with the research of (Diaz-de Mera, 2017). In addition, higher values of wind speed produces also a relative risk in Algeciras. Nevertheless, in the case of PM_10_, the highest values of wind speed cause protection leverage in this city, just the reverse than in La Línea station, where higher values of wind direction and higher temperatures act as protection. Admittedly, PM_10_ pollutant is spread from Algeciras to La Línea through wind speed.

According to trend analysis, NO_2_ pollutant presents similarities between Algeciras and La Línea stations. In the case of SO_2_, the similarity is between Algeciras and Los Barrios station and with regard to PM_10_ it can be measured indistinctly in Algeciras-Los Barrios, El Rinconcillo-La Línea or El Zabal-La Línea stations. These results, in general, are confirmed with the regression models.

According to the OECD, outdoor air pollution could cause 6–9 million premature deaths a year by 2060 and cost 1% of global GDP^9^—around USD 2.6 trillion annually—as a result of sick days, medical bills and other issues.

In accordance with recent estimates by the WHO, exposure to air pollution is thus a more important risk factor for major diseases. New tools such the one presented in this work have proven to be an effective tool in avoiding hazardous situations. Preventing potentially health risk events helps citizens in preventing morbidity and premature mortality, one of the targets under Sustainable Development Goal (objective 3) on good health and promoting wellbeing.

To summarise the above, an air pollution modelling approach based on different perspectives (descriptive, predictive, and prescriptive) was performed on this singular area using different statistical methods. The results obtained can be used as a virtual sensor in the case of sensor failures and also as a support decision tool for institutions and citizens to prevent peak-situations. Furthermore, this proposed approach could be used in different regions or scenarios in future researches.

## Footnotes

1 ECA = Emission Control Areas.

2 IMO = International Maritime Organization. http://www.imo.org/.

3 AQI = Air Quality Index.

4 https://www.apba.es/datos-tecnicos.

5 *Ɛ* $i$ is the difference between the observed and the predicted values.

6 United Nations Economic Commission for Europe.

7 World Health Organization.

8 Organisation for Economic Co-operation and Development.

9 Gross Domestic Product.
